# Severity of Subjective Cognitive Complaints and Worries in Older Adults Are Associated With Cerebral Amyloid-β Load

**DOI:** 10.3389/fnagi.2021.675583

**Published:** 2021-08-02

**Authors:** Claudia Schwarz, Catharina Lange, Gloria S. Benson, Nora Horn, Katharina Wurdack, Mathias Lukas, Ralph Buchert, Miranka Wirth, Agnes Flöel

**Affiliations:** ^1^Department of Neurology, Charité - Universitätsmedizin Berlin, Corporate Member of Freie Universität Berlin and Humboldt-Universität zu Berlin, Berlin, Germany; ^2^NeuroCure Clinical Research Center, Charité - Universitätsmedizin Berlin, Corporate Member of Freie Universität Berlin and Humboldt-Universität zu Berlin, Berlin, Germany; ^3^Department of Nuclear Medicine, Charité - Universitätsmedizin Berlin, Corporate Member of Freie Universität Berlin and Humboldt-Universität zu Berlin, Berlin, Germany; ^4^Siemens Healthcare GmbH, Berlin, Germany; ^5^Department for Diagnostic and Interventional Radiology and Nuclear Medicine, University Hospital Hamburg-Eppendorf, Hamburg, Germany; ^6^German Center for Neurodegenerative Diseases (DZNE) Site: Dresden, Dresden, Germany; ^7^Department of Neurology, University Medicine Greifswald, Greifswald, Germany; ^8^German Center for Neurodegenerative Diseases (DZNE) Site: Greifswald, Greifswald, Germany

**Keywords:** subjective cognitive decline, preclinical Alzheimer's disease, ^18^F-florbetaben PET, amyloid, biomarkers, cognitively normal

## Abstract

Subjective cognitive decline (SCD) is considered an early risk stage for dementia due to Alzheimer's disease (AD) and the development of pathological brain changes, such as the aggregation of amyloid-beta (amyloid-β) plaques. This study evaluates the association between specific features of SCD and cerebral amyloid-β load measured by positron emission tomography (PET) with ^18^F-florbetaben in 40 cognitively normal older individuals. Global amyloid-β, as well as regional amyloid-β load for the frontal, temporal, parietal, and cingulate cortex, was quantified. Specific features of SCD, such as subjective cognitive complaints and worry, were assessed using the 39-item Everyday Cognition Scales and the 16-item Penn State Worry Questionnaire. Spearman's rank partial correlation analyses, adjusted for age and apolipoprotein E ε4 status, were conducted to test the associations between specific features of SCD and cerebral amyloid-β load. The severity of subjective cognitive complaints in everyday memory and organization was positively correlated with amyloid-β load in the frontal cortex. In addition, the severity of subjective cognitive complaints in everyday planning was positively correlated with amyloid-β load in the parietal cortex. Higher levels of worry were associated with higher amyloid-β load in the frontal cortex. After correction of the PET data for partial volume effects, these associations were reduced to trend level. In conclusion, the severity of subjective cognitive complaints and the level of trait worry were positively associated with cortical amyloid-β burden, particularly in the frontal and parietal cortex. Further studies are required to elucidate the direction of these associations in order to develop strategies to prevent amyloid deposition and cognitive decline.

## Introduction

Subjective cognitive decline (SCD) is characterized by a persistent self-perceived deterioration of cognitive performance in cognitively normal older people, which occurs in the absence of objective cognitive impairment, and is unrelated to an acute event (Jessen et al., 2014a, 2020). Several studies indicate SCD as an at-risk stage for future cognitive decline and eventual clinical progression to mild cognitive impairment (MCI) and dementia due to Alzheimer's disease (AD) (Jessen et al., 2014b, 2020; Koppara et al., 2015; Wolfsgruber et al., 2016).

Several characteristics of SCD, so-called “SCD-plus features,” have been identified and found to be associated with an increased risk of cognitive decline and AD (Jessen et al., 2010, 2014a,b). One SCD-plus feature that has been well described in several studies is the persistent feeling of cognitive worsening in memory performance. Another well-described SCD-plus feature is the presence of worries (concerns) associated with the subjective feeling of cognitive deterioration. The manifestation of distinct worries about self-perceived cognitive decline over a longer period of time also reflects the presence of specific psychological traits associated with cognitive decline and AD-related pathologies, such as repetitive negative thinking (RNT) (Marchant et al., 2020; Schlosser et al., 2020). Both the persistent feeling of cognitive decline in memory performance and associated worries (concerns) are considered as SCD-plus features, increasing the likelihood that individuals with SCD will develop AD.

Strong evidence exists for the presence of early pathological brain alterations associated with AD in older individuals with SCD, including the accumulation of amyloid-beta (amyloid-β) plaques in the cortex (Jessen et al., 2020). Previous studies provide evidence for positive associations of cerebral amyloid-β load, measured by positron emission tomography (PET), with the severity of subjective cognitive complaints, particularly with respect to memory performance (Amariglio et al., 2012; Perrotin et al., 2012). In addition, lower amyloid-β 42 concentration measured in cerebrospinal fluid (CSF) of cognitively normal older persons was found to be associated with several SCD-plus features, such as a self-reported decrease in memory and language performance and associated worries (concerns) (Miebach et al., 2019). A recent study showed associations of cognitive decline-related worries with global as well as regional amyloid-β load (including the frontal, parietal, temporal, and cingulate lobe) in older individuals with SCD (Verfaillie et al., 2019). Moreover, a longitudinal study (2–6 years of follow-up data) has shown that subjective cognitive complaints along with the accumulation of cerebral amyloid-β predict a faster decline in cognitive abilities in normal older adults (Amariglio et al., 2018).

Previous studies, however, have primarily focused on global amyloid-β aggregation or in a composite of AD-vulnerable regions of interest (ROIs) in older individuals, mostly dichotomized by the presence or absence of amyloid-β accumulation, assessing mainly the association with self-perceived memory decline. In this study, we aimed to further investigate the phenotype of SCD by evaluating the association between specific features of SCD, including the severity of subjective cognitive complaints in different domains and level of worry, and cerebral global amyloid-β load as well as in predefined ROIs by scanning cognitively normal older individuals on a hybrid scanner system for simultaneous PET and magnetic resonance imaging (MRI). The results were interpreted for both uncorrected PET images and PET images corrected for partial volume effects (PVE), increasing the validity of the results.

## Methods

### Study Design

Cross-sectional data from 40 cognitively normal older individuals, including 30 participants with SCD from the SmartAge study [clinicaltrials.gov: NCT03094546 (Wirth et al., 2019)] and 10 healthy controls (HC), who were recruited as a control group within the SmartAge trial, were used for this analysis. The protocol was carried out at the NeuroCure Clinical Research Center, Charité - Universitätsmedizin Berlin, and encompassed neuropsychological and behavioral testing, a medical examination including blood sampling, as well as the assessment of cerebral amyloid-β PET.

The study has been approved by the responsible institutional review board (Ethics Committee of the Charité - Universitätsmedizin Berlin, Germany) as well as the federal radiation protection authority (Bundesamt für Strahlenschutz) and was carried out in compliance with institutional ethical standards and the Declaration of Helsinki. All study participants provided written informed consent.

### Participants

Cognitively normal older individuals, aged between 60 and 90 years and fluent German speakers, were recruited both from healthcare institutions and through advertisements in the general population. Study eligibility was assessed during study enrollment, through a telephone screening and an on-site screening. The exclusion criteria for potential study participation comprised severe or untreated medical, psychiatric, and neurological disorders (including the diagnosis of MCI or dementia) as well as malignancies, alcohol dependency, and drug abuse. During the on-site screening, participants had to show a Mini-Mental State Examination (MMSE) score ≥26, an objective cognitive performance within −1.5 standard deviation (SD) of age-adjusted norms in selected neuropsychological tests, an absence of deficits in selected items of the Instrumental Activities of Daily Living Scale, and a score ≤10 in the 15-item Geriatric Depression Scale (GDS) [detailed information is provided in the SmartAge study protocol (Wirth et al., 2019)]. The classification of study participants in SCD and HC was based on established guidelines and encompassed the fulfillment of the following criteria for a diagnosis of SCD: (1) the presence of SCD for at least six months, (2) related worries (concerns), and (3) previous consultation or consideration to consult a doctor due to these symptoms (Jessen et al., 2014a). Participants were considered as HC for study participation if such subjective cognitive deterioration was considered as no severe worries (concerns) or not present. For this analysis, both diagnostic groups were combined as cognitively normal older individuals to increase the sample size as well as the variance of self-perceived cognitive decline and worry in cognitively healthy older adults.

### Acquisition of Neuropsychological, Behavioral, and Genetic Data

All participants completed an extensive neuropsychological test and questionnaire battery, administered by trained assessors. For assessing subjective cognitive complaints, the self-reported 39-item Everyday Cognition Scales (ECog-39) (Farias et al., 2008) was applied. This questionnaire measures subjective changes in cognitively mediated functional abilities compared to 10 years earlier by capturing six cognitive domains, namely, everyday memory, language, visuospatial abilities, planning, organization, and divided attention. Scoring was based on a continuous scale of 4 response options ranging from “better or no change” to “consistently much worse” as well as an additional response option labeled “I don't know,” which was treated as missing data. The average score was calculated by the total amount of answered questions for all 39 items (i.e., total score) and for each cognitive domain separately.

The trait of worry was assessed by the self-reported 16-item Penn State Worry Questionnaire (PSWQ) (Stöber, 1995; Glöckner-Rist and Rist, 2014). This Likert-type scale is designed to measure the frequency, intensity, uncontrollability, and other characterizing features of worry. The 16 items are rated on a 5-point scale ranging from “not at all typical of me” to “very typical of me.” The individual item scores are summed to provide a total score that ranges from 16 to 80, with higher scores indicating more severe worry and general anxiety (Stanley et al., 2003; Webb et al., 2008).

As part of the standard medical examination, blood sampling was performed on all participants. The single nucleotide polymorphism in apolipoprotein E (APOE) was analyzed for a detailed characterization of the participants regarding the APOE ε4 status. The purification of *in vitro* DNA was performed using QIAamp DNA Blood Mini Kit (QIAGEN, Hilden, Germany), followed by a DNA quality check using NanoDrop photometer (Thermo Fisher Scientific, Wilmington, DE, USA), and stored at −80°C until further processing and analysis, at the NeuroCure Clinical Research Center, Charité - Universitätsmedizin Berlin. The standardized preanalytic procedures were set up and assured by the NeuroHub biomarker management platform and LabVantage software (Schwarz et al., 2018; Wirth et al., 2019). The APOE genotypes (i.e., rs429358 and rs7412) were determined by TaqMan assays in the laboratory of Prof. Dr. Dan Rujescu (University of Halle, Germany) according to previously described procedures (O'Dwyer et al., 2012).

### Acquisition and Preprocessing of Imaging Data

Cerebral amyloid-β PET and structural MR images used in this study were acquired simultaneously at a 3 Tesla PET/MR system (Biograph mMR, Siemens Healthcare GmbH, Erlangen, Germany). Image processing was performed with routines of the Statistical Parametric Mapping (SPM) package (Wellcome Trust Centre for Neuroimaging, London, UK, version SPM12, www.fil.ion.ucl.ac.uk/spm) using MATLAB (The MathWorks, Natick, MA, USA, version R2013b).

#### MRI Images

The high-resolution T1-weighted MR images were acquired by using a sagittal three-dimensional magnetization-prepared rapid gradient echo (3D-MPRAGE) sequence with an approximate repetition time = 2,400 ms, minimum full echo time, inversion time = 900 ms, and flip angle of 8° resulting in 1 mm isotropic images. DICOM-to-NifTi conversion was performed with mcverter (https://lcni.uoregon.edu/downloads/mriconvert/mriconvert-and-mcverter). For the segmentation of brain tissue into gray matter, white matter, and CSF, the unified segmentation algorithm of SPM12 was deployed with default parameters (Ashburner and Friston, 2005), except that the image data was resampled to 2 × 2 × 2 mm (Herron et al., 2012). Normalization to the Montreal Neurological Institute (MNI) template space was performed using diffeomorphic anatomical registration through exponentiated lie algebra with default parameters and registration to “existing templates” (Ashburner, 2007) using the IXI550 templates (CAT12 toolbox, http://www.neuro.uni-jena.de/wordpress/vbm/).

#### Amyloid PET Images

Cerebral amyloid-β PET was acquired by using ^18^F-florbetaben (FBB, Neuraceq^®^, Life Radiopharma Berlin GmbH, Berlin, Germany). A late PET scan of the brain with 20 min duration was acquired 90.8 ± 3.4 min after the intravenous administration of 288.6 ± 16.3 MBq FBB according to common guidelines (Barthel et al., 2016). The PET data were reconstructed into 20 frames each of 1 min duration using the ordered subset expectation maximization reconstruction algorithm provided by the scanner manufacturer (i.e., three iterations, 21 subsets, no filter), whereas the μ-map for attenuation correction was calculated using the algorithm proposed by Cabello et al. (2015).

The DICOM-to-NifTi conversion was performed with dcm2nii (https://people.cas.sc.edu/rorden/mricron/dcm2nii.html). Post-reconstruction filtering was performed by a three-dimensional Gaussian kernel with full width at half maximum (FWHM) of 2 mm since no filtering was applied during the iterative reconstruction of the PET data. The image processing included post-acquisition and post-reconstruction inter-frame motion correction as described by Lange et al. (2015). A motion-corrected static uptake image was obtained by summing all frames after realignment and, finally, co-registered to the individual T1-weighted MRI as well as spatially normalized to MNI space using the T1-based deformation fields (see the “MRI images” section).

In addition, correction of PVE was performed using the Müller-Gärtner method (Müller-Gärtner et al., 1992) as implemented in the PETPVE12 toolbox developed and validated by Gonzalez-Escamilla et al. (2017). The individual tissue probability maps were used as input data for PVE correction, and a 6 mm FWHM was deployed as reconstructed spatial resolution of the PET images. FBB standard uptake value ratios (SUVRs) were calculated for the four commonly used ROIs, i.e., the frontal, temporal, parietal, and cingulate cortex, by scaling the average ROI standard uptake value (SUV) to the average ROI SUV of a reference region (i.e., the whole cerebellum). We followed a method described by the Alzheimer's Disease Neuroimaging Initiative (ADNI) PET core team (Landau and Jagust, 2014), i.e., the non-corrected SUV of the whole cerebellum as defined by the Desikan atlas was used to adjust both the non-corrected and the PVE-corrected cortical SUV data (Desikan et al., 2006). The cortical regions were composed of Desikan subregions (i.e., frontal: 18, cingulate: 8, temporal: 4, and parietal: 8) using a weighted SUV average to account for varying volumes. Finally, the cortical composite measure [i.e., composite SUVR (cSUVR)], indicating global amyloid-β load, was calculated by creating a conventional (i.e., non-weighted) average across all cortical SUVRs.

Based on the visual inspection of the FBB PET images by two experienced and independent raters, scans were judged as amyloid positive or amyloid negative according to the common guidelines for amyloid PET (Barthel et al., 2016). The visual assessment focused on amyloid-β load in lateral temporal, frontal, posterior cingulate, and parietal cortex, in line with the regions used for semi-quantification. If the amyloid-β load in at least one region was determined as “moderate” or “pronounced,” the whole scan was rated as amyloid positive. Discordant readings were present in two participants. In these cases, a consensus reading between both raters was conducted by adding the 3D-MPRAGE to the PET images (i.e., side-by-side reading).

### Statistical Analysis

The statistical analyses of sample characteristics and correlation analyses were performed with the IBM SPSS Statistics software version 25.0 (IBM Corp, Released in 2017; IBM SPSS Statistics for Microsoft, version 25.0; IBM Corp., Armonk, NY, USA), and figures were created in R version 4.0.3 (Team, 2013) using the package ggpubr (Kassambara, 2018). The significance level was set at α = 0.05. The characteristics of study participants were reported descriptively using mean, SD, percentage, and range for each feature, if applicable. The associations between specific features of SCD, such as subjective cognitive complaints (i.e., total mean and in different domains) and worry, and global/regional cerebral amyloid-β load were tested by the Spearman's rank partial correlation analyses, adjusted for age and APOE ε4 status. Given the exploratory nature of the analyses, no correction for multiple comparisons was applied; thus, all analyses should be interpreted as non-confirmative.

## Results

### Characteristics of Participants

The characteristics of the participants, including demographics, cognition and mental health, genetics, and information on cerebral amyloid-β load, are presented for the total sample as well as for the HC and SCD groups separately in Table 1. The mean age of the total sample was 68 years, 18 participants (45%) were women, and the mean years of education were 16. The participants demonstrated normal cognition on the MMSE with a mean score of 29.2 and no evidence of impaired mental health based on a mean GDS score of 1.5, a mean ECog-39 total score of 1.6, and a mean PSWQ total score of 38.7. In total, 10 participants of the SCD group were identified as APOE ε4 carriers, and the assessment of FBB PET revealed 8 amyloid-positive participants by visual assessment (i.e., 7 in the SCD and 1 in the HC group) and a mean cortical cSUVR of 1.1 in the total sample.

**Table 1:** Characteristics of study participants.

	**All (*n* = 40)**	**HC (*n* = 10)**	**SCD (*n* = 30)**
**Demographics**			
Females [*n*] (%)	18 (45.0)	3 (30.0)	15 (50.0)
Age [years]	68 (6), 60–83	72 (8), 63–83	67 (4), 60–76
Education [years]	16 (3), 11–23	15 (2), 11–19	17 (3), 11–23
**Cognition and mental health**			
MMSE [score]	29.2 (0.8), 27.0–30.0	29.2 (1.0), 27.0–30.0	29.2 (0.7), 28.0–30.0
ECog-39 [score]	1.6 (0.4), 1.1–2.7	1.2 (0.1), 1.1–1.4	1.7 (0.4), 1.1–2.7
GDS [score]	1.5 (1.2), 0–4.0	0.6 (0.7), 0–2.0	1.7 (1.1), 0–4
PSWQ [score]	38.7 (11.1), 16.0–59.0	31.3 (7.7), 19.0–46.0	41.1 (11.0), 16.0–59.0
**Genetics**			
APOE ε4 carrier [*n*] (%)	10 (25.0)	0 (0)	10 (33.3)
**FBB PET**			
Amyloid positive [*n*] (%)	8 (20.0)	1 (10.0)	7 (23.3)
cSUVR	1.1 (0.2), 0.9–1.9	1.1 (0.2), 1.0–1.5	1.1 (0.2), 0.9–1.9

*The data are given, if applicable, as mean (SD or %) and range for all participants as well as for each group (HC and SCD) separately. The amyloid positive rating was based on the visual assessment of FBB PET images. APOE ε4, apolipoprotein E ε4; cSUVR, composite standard uptake value ratio; ECog-39, 39-item Everyday Cognition Scales; FBB PET, ^18^F-florbetaben PET; GDS, Geriatric Depression Scale; HC, healthy controls; MMSE, Mini-Mental State Examination; PSWQ, Penn State Worry Questionnaire; SCD, subjective cognitive decline; SD, standard deviation*.

### Cerebral Amyloid-β Load, Subjective Cognitive Complaints, and Worry

**Figure 1:**
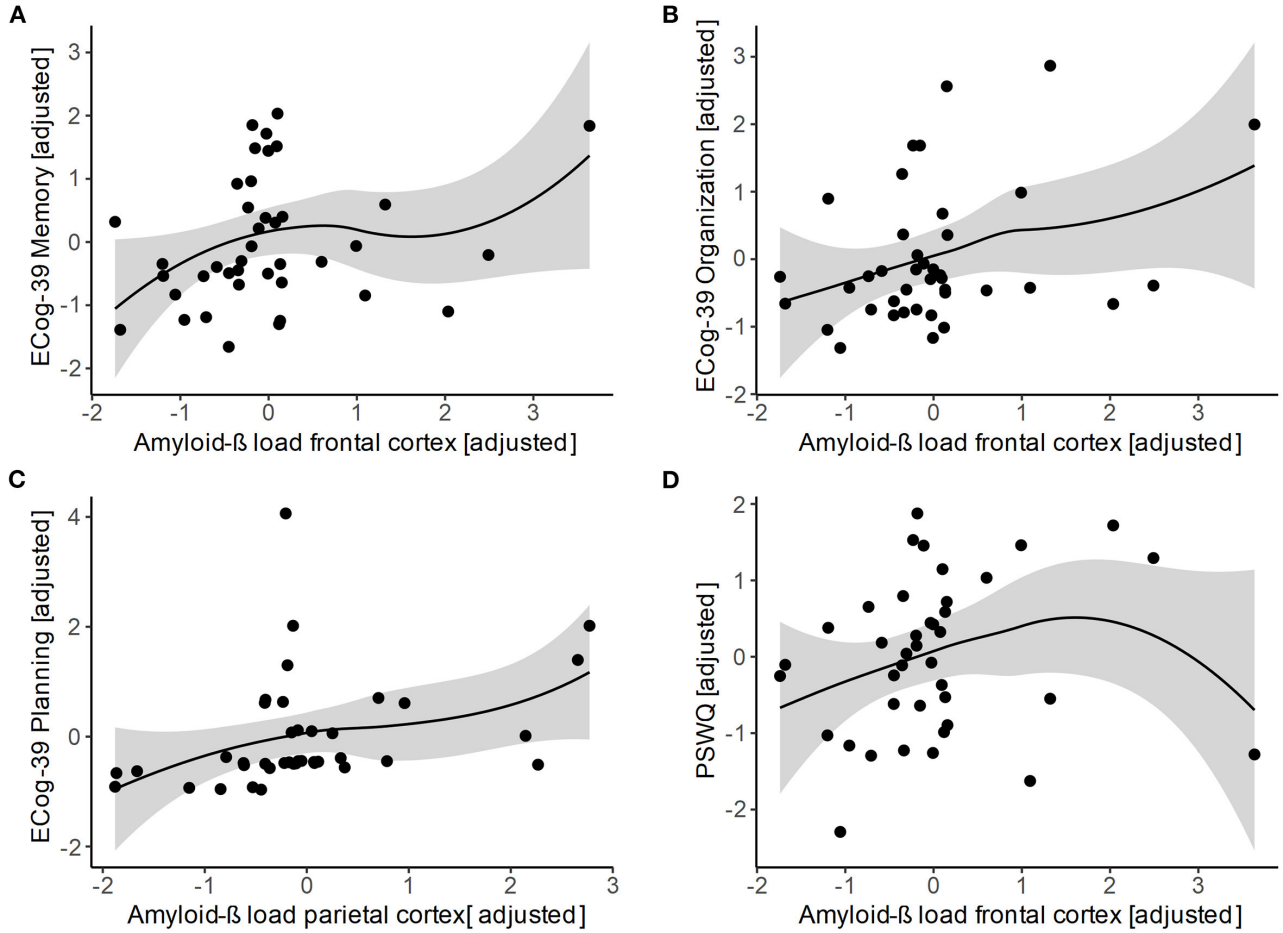
Scatter plots of the associations of regional amyloid-β load in the frontal and parietal cortex with subjective cognitive complaints in **(A)** memory, **(B)** organization, **(C)** planning, as well as with **(D)** level of worry. All variables are standardized residuals after adjusting for age and APOE ε4 status. Regional amyloid-β load was not corrected for partial volume effects. Loess regression lines with 95% CIs were added. Amyloid-β, amyloid-beta; APOE ε4, apolipoprotein E ε4; ECog-39, 39-item Everyday Cognition Scales; PSWQ, Penn State Worry Questionnaire.

The investigation of the relationship between global and regional amyloid-β load and the severity of subjective cognitive complaints (i.e., total and in different cognitive domains) as well as the level of worry is shown in Table 2; Figure 1. We observed a positive association of subjective complaints in planning with amyloid-β load in the parietal cortex (*p* = 0.042). In addition, amyloid-β load in the frontal cortex was positively correlated with subjective complaints in memory (*p* = 0.034) and organization (*p* = 0.047). Moreover, we found a positive association between the level of worry and the amyloid-β load in the frontal cortex (*p* = 0.031). No significant correlations were observed for the other tested associations.

**Table 2:** Association of amyloid-β load, subjective cognitive complaints, and worry.

	**Composite SUVR**		**Frontal SUVR**		**Temporal SUVR**		**Parietal SUVR**		**Cingulate SUVR**	
	**rho**	***p*-value**	**rho**	***p*-value**	**rho**	***p*-value**	**rho**	***p*-value**	**rho**	***p*-value**
**ECog-39**										
Total score [mean]	0.164	0.326	0.277	0.092	0.033	0.843	0.119	0.476	0.088	0.598
Memory	0.194	0.244	0.345	**0.034**	−0.002	0.990	0.129	0.440	0.132	0.428
Language	0.069	0.681	0.164	0.326	−0.028	0.869	0.085	0.613	0.014	0.933
Visuospatial abilities	0.201	0.227	0.197	0.236	0.104	0.533	0.151	0.366	0.163	0.329
Planning	0.281	0.087	0.272	0.098	0.192	0.249	0.332	**0.042**	0.156	0.351
Organization	0.313	0.055	0.325	**0.047**	0.179	0.283	0.205	0.216	0.227	0.170
Divided attention	0.143	0.390	0.234	0.158	0.047	0.778	0.088	0.600	0.062	0.712
**PSWQ**										
Total score [sum]	0.260	0.115	0.351	**0.031**	0.274	0.096	0.042	0.801	0.176	0.290

*Rho- and p-values were estimated from the Spearman's rank partial correlation analyses, adjusted for age and APOE ε4 status. Global and regional amyloid-β load were not corrected for partial volume effects. P-values ≤ 0.05 are indicated in bold. Amyloid-β, amyloid-beta; APOE ε4, apolipoprotein E ε4; ECog-39, 39-item Everyday Cognition Scales; PSWQ, Penn State Worry Questionnaire; SUVR, standard uptake value ratio*.

All significant associations did not survive the PVE correction of the PET data. However, trend levels for positive associations of amyloid-β load in the frontal cortex with subjective complaints in planning (*p* = 0.096) and divided attention (*p* = 0.053) as well as with the level of worry (*p* = 0.065) were observed. In addition, amyloid-β load in the parietal cortex and subjective complaints in planning were positively associated on a trend level (*p* = 0.081).

### Exploratory Analyses

In *post hoc* analyses, we assessed the associations between severity of subjective cognitive complaints and level of worry with hippocampal volume (i.e., adjusted for total intracranial volumes). No significant relationships were observed (i.e., all rho values ≤0.255 and all *p*-values ≥0.128).

## Discussion

In this study, we investigated the association between specific features of SCD, including subjective cognitive complaints and worry, and global as well as regional cerebral amyloid-β load in cognitively normal older individuals, showing that the severity of self-experienced decline in specific cognitive domains and the level of worry are positively associated with regional amyloid-β burden. These associations were found predominantly in the frontal as well as, but to a lesser extent, in the parietal cortex for the non-PVE-corrected PET images. The correction of PVE resulted in a decrease in the significance of the tested associations to trend level.

The severity of subjective complaints in specific cognitive domains, such as memory, organization, and planning, and level of worry were positively associated with regional cerebral amyloid-β load. This result is in line with the recent study of Miebach et al. (2019), showing, among others, associations of self-experienced decline in memory function as well as decline-related worries (concerns) with lower amyloid-β 42 concentrations measured in the CSF of cognitively normal individuals. In addition, our results are supported by a previous study of Amariglio et al. (2012), demonstrating associations of amyloid-β load in a composite of AD-vulnerable regions with subjective complaints in a memory composite score (including the ECog-39 memory domain) and everyday planning (assessed using the ECog-39). However, in their study, subjective complaints of the other cognitive domains assessed by the ECog-39 revealed no association with amyloid-β load. Moreover, the association of worry with amyloid-β load is in line with a recent study showing a positive association of RNT with global amyloid-β load in healthy older participants (Marchant et al., 2020). In contrast to our measure of anxiety-related pathological trait of worry, the cognitive process RNT encompasses future-related worry and past-related rumination. However, RNT was shown to be related to anxiety and depression symptoms. Our results suggest that in addition to cognitive decline-related worries and RNT, the general anxiety-related trait of worry may be associated with AD-related pathology and cognitive decline. This finding strengthens the assumption that several psychoaffective factors, such as worry and anxiety, and subjective cognitive complaints might be interrelated in older individuals with SCD (Comijs et al., 2002; Hill et al., 2016; Kuhn et al., 2019). Moreover, psychoaffective factors and SCD might, in combination, exacerbate the risk for future cognitive decline and clinical progression to AD dementia (Liew, 2020). Data from an observational cohort study suggested that higher levels of worry, as assessed by the PSWQ, are associated with worse learning and memory performance in older individuals and may also predict future cognitive decline (Pietrzak et al., 2012). Thus, our findings indicate that subjective cognitive complaints and worries may reflect regional cerebral amyloid-β load in cognitively normal older individuals; however, the direction of these associations remains unclear.

The associations of subjective cognitive complaints and worry with amyloid-β load were region-dependent and observed predominantly in AD-vulnerable regions, such as the frontal and parietal cortex. This finding is supported by Perrotin et al. (2012), who revealed a positive association of subjective memory complaints with regional amyloid-β load in the right medial prefrontal cortex and anterior cingulate cortex as well as in the posterior cortices. The assessment of subjective memory complaints for these associations was based on a global question regarding the memory performance of the participants relative to other people of the same age. In contrast, Verfaillie et al. could not provide evidence for an association of global as well as regional amyloid-β load in frontal, temporal, parietal, and cingulate cortical regions with the severity of cognitive complaints (Verfaillie et al., 2019). However, they supported our finding of an association of worry with amyloid-β load in the frontal cortex by showing associations of decline-related worries with global as well as regional amyloid-β load (e.g., in the frontal lobe) in older individuals with SCD. In our study, self-experienced decline in everyday memory and organization, as well as level of worry, were predominantly related to amyloid-β load in the frontal cortex, a region involved in many cognitive processes including memory formation, social cognition, and cognitive control (Buckner et al., 1999; Ridderinkhof et al., 2004; Amodio and Frith, 2006). Furthermore, self-perceived decline in the executive function domain of everyday planning is associated with amyloid-β load in the parietal cortex, a region known to be involved in planning (Andersen and Cui, 2009). Interestingly, these regions are also often linked to self-reflective thoughts and metacognitive functions (Johnson et al., 2002; Schmitz et al., 2004). In addition, the frontal and parietal cortex are strongly interconnected and considered as core regions of the default mode network that is affected early in AD by amyloid-β accumulation (Palmqvist et al., 2017). Thus, our findings suggest that subjective cognitive complaints and worries may represent the first clinical manifestations of AD.

Our approach of analyzing the PET data with and without PVE correction resulted in a decrease in significance of the tested associations to trend level when correcting for PVE. This might be due to the fact that subjects of our sample did not show global cortical atrophy beyond normal aging, resulting in only a small benefit of PVE correction. In addition, PVE correction methods highly depend on several model assumptions that may hold true only in the ideal imaging data (Erlandsson et al., 2012), which potentially led to a reduced effect size by noise amplification of global SUVR. Therefore, we assumed that the detrimental effect of noise amplification outweighed the overall benefit of PVE correction in this sample.

The limitations of our study include, first, the relatively small sample size, which is, however, comparable to other studies using FBB PET (Li et al., 2021). Nevertheless, it will be important to test in future studies whether such findings generalize to the aging population. Second, the correction of amyloid PET for PVE may not have improved the quality of our data. However, the usefulness of PVE correction in amyloid PET has been extensively evaluated in previous studies (Schmidt et al., 2015; Matsubara et al., 2016; Gonzalez-Escamilla et al., 2017), demonstrating that PVE correction generally increases the averaged difference of global SUVR between amyloid-β-positive and amyloid-β-negative subjects and, therefore, boosts the statistical power of correlation analyses.

## Conclusion

The findings of this study indicate that the manifestation of subjective cognitive complaints and worry in cognitively normal older individuals may reflect the presence of amyloid-β-related pathology and may be a clinical indicator of early AD. Further studies are required to elaborate on the direction of these associations in order to prevent amyloid deposition in these regions as well as future cognitive decline.

## Data Availability Statement

Anonymized data will be made available to the scientific community upon request.

## Ethics Statement

The study involving human participants was reviewed and approved by Ethics Committee of the Charité Universitätsmedizin Berlin, Germany. The participants provided their written informed consent to participate in this study.

## Author Contributions

CS, CL, GB, ML, RB, MW, and AF contributed substantially to the overall design and conceptualization of the study. CS, CL, GB, NH, and KW took part in the implementation and conduction of the study. CS, CL, GB, RB, MW, and AF performed the analysis and interpretation of the data. CS, CL, RB, MW, and AF drafted the manuscript. All authors took part in revising the manuscript for content and approved the final version of the manuscript.

## Conflict of Interest

AF has obtained consulting fees from Bayer and Biogen Idec., honoraria for oral presentations from Novartis, Roche, Daiichi Sankyo, Biogen Idec, and DrSchär AG, as well as royalties from the book *Alzheimer-Unabwendbares Schicksal?* ML was employed by the company Siemens Healthcare GmbH. The remaining authors declare that the research was conducted in the absence of any commercial or financial relationships that could be construed as a potential conflict of interest.

## Publisher's Note

All claims expressed in this article are solely those of the authors and do not necessarily represent those of their affiliated organizations, or those of the publisher, the editors and the reviewers. Any product that may be evaluated in this article, or claim that may be made by its manufacturer, is not guaranteed or endorsed by the publisher.

## Abbreviations

AD: Alzheimer's disease
ADNI: Alzheimer's disease neuroimaging initiative
APOE: apolipoprotein E
amyloid-β: amyloid-beta
CSF: cerebrospinal fluid
cSUVR: composite standard uptake value ratio
ECog-39: 39-item Everyday Cognition Scales
FBB: ^18^F-florbetaben
FWHM: full width at half maximum
GDS: Geriatric Depression Scale
GM: gray matter
HC: healthy controls
MCI: mild cognitive impairment
MMSE: Mini-Mental State Examination
MNI: Montreal Neurological Institute
MRI: magnetic resonance imaging
PET: positron emission tomography
PSWQ: Penn State Worry Questionnaire
PVE: partial volume effects
RNT: repetitive negative thinking
ROI: region of interest
SCD: subjective cognitive decline
SUV: standard uptake value
SUVR: standard uptake value ratio
WM: white matter
3D-MPRAGE: three-dimensional magnetization-prepared rapid gradient echo.
